# Robot-assisted gait training alleviates the symptoms of acute ischemic stroke via regulating gut microbiota composition and function

**DOI:** 10.3389/fcimb.2026.1810501

**Published:** 2026-04-29

**Authors:** Haijie Liu, Bo Han, Hui Yao, Junjie Wang, Fang Xv, Yujie Xv, Meng Zhang, Ming Lin, Zeguang Li, Sen Wang, Yuting Zhang, Juan Gao, Yanming Zhang, Peng Zhang

**Affiliations:** 1 Department of Neurology, Xuanwu Hospital Capital Medical University, National Center for Neuro-logical Disorders, Beijing, China; 2 Beijing Municipal Geriatric Medical Research Center, Beijing, China; 3 Department of Neurology, Baoding No. 1 Central Hospital, Baoding, Hebei, China; 4 Department of Rehabilitation Medicine, Xuanwu Hospital Capital Medical University, National Center for Neuro-logical Disorders, Beijing, China; 5 Tianjin Key Laboratory of Life and Health Detection, Life and Health Intelligent Research Institute, Tianjin University of Technology, Tianjin, China

**Keywords:** 16S rRNA sequencing, acute ischemic stroke, dysbiosis, gut microbiota, robot-assisted gait training

## Abstract

**Background:**

Robot-assisted gait training (RAGT) improves motor recovery after stroke, yet its biological mechanisms remain poorly understood. Increasing evidence suggests that gut microbiota dysbiosis contributes to stroke pathology and recovery. *Objective*. To investigate whether RAGT alleviates acute ischemic stroke (AIS) symptoms by modulating gut microbiota composition and function.

**Methods:**

Forty-seven newly diagnosed AIS patients were enrolled and assigned to a RAGT group (IG, *n* = 24) or a conventional gait training group (CG, *n* = 23). Fecal samples were collected after the intervention, and functional outcomes were assessed at 90 days. Gut microbiota composition and predicted function were analyzed using 16S rRNA gene sequencing.

**Results:**

Compared with CG patients, IG patients demonstrated significantly improved clinical outcomes. RAGT partially reversed stroke-associated gut dysbiosis, characterized by increased microbial richness and evenness. Beneficial taxa, including *Lachnospiraceae*, *Ruminococcaceae*, and *Oscillospiraceae*, were enriched, whereas *Enterobacteriaceae* and *Bifidobacteriaceae* were reduced. Functional prediction revealed decreased pathogenic potential of the gut microbiota in the IG, which correlated with improved AIS outcomes.

**Conclusion:**

Poststroke RAGT was associated with alteration of gut microbiota composition and function, suggesting a novel microbiota-mediated mechanism underlying its neurorehabilitative benefits in AIS.

## Introduction

Stroke remains a major global health burden and is one of the leading causes of long-term disability worldwide (Parr et al., 2017). Despite advances in acute management, only a small proportion of stroke survivors achieve full functional recovery. Most patients experience persistent impairments, including motor dysfunction, sensory deficits, cognitive decline, and psychological disturbances (Hornby et al., 2016; Cramer, 2020). Among these sequelae, lower limb motor dysfunction is particularly prevalent, affecting approximately two-thirds of stroke survivors and resulting in substantial limitations in essential activities such as sitting, transferring, and ambulation. Poststroke gait abnormalities including reduced gait speed, impaired balance, decreased endurance, and increased fall risk, further compromise independence and quality of life (Duncan et al., 2011; Lee et al., 2019).

Restoration of independent walking is therefore a central goal of stroke rehabilitation, as it directly influences activities of daily living (ADLs), mobility, and social participation (Perry et al., 1995). In recent years, lower limb rehabilitation robots have been increasingly integrated into clinical practice. RAGT enables early, repetitive, task-specific, and safe walking practice, which are key principles of neurorehabilitation. Clinical and cohort studies have demonstrated that RAGT improves walking efficiency, balance, and gait speed in stroke patients, particularly when initiated early in recovery (Bruni et al., 2018; Zheng et al., 2019; Moucheboeuf et al., 2020). However, while the functional benefits of RAGT are well documented, the biological mechanisms underlying its therapeutic effects remain incompletely understood.

Physical exercise is increasingly recognized as a non-pharmacological modulator of gut microbiota composition and function. Both animal and human studies indicate that exercise enhances microbial diversity and promotes the expansion of beneficial, short-chain fatty acid–producing bacteria (Matsumoto et al., 2008; Clarke et al., 2014). Nevertheless, clinical evidence regarding the effects of poststroke rehabilitation exercise, particularly structured gait training, on stroke-induced gut microbiota dysbiosis remains limited. Whether RAGT exerts part of its neurorehabilitative benefits through modulation of the gut microbiota has not yet been systematically explored.

In this study, we aimed to investigate the effects of RAGT on gut microbiota composition and function in patients with AIS. Furthermore, we sought to determine whether RAGT-associated alterations in the gut microbiota are linked to improved functional recovery, thereby providing novel mechanistic insights into the microbiota-mediated effects of rehabilitation after stroke.

## Methods

### Study design and participants

This prospective, non-randomized controlled cohort study was conducted at the Department of Neurology, Xuanwu Hospital, Capital Medical University, and the Department of Neurology, Baoding First Central Hospital between September and December 2022. Patients newly diagnosed with AIS within 7 days of symptom onset were screened for eligibility.

Eligible participants were required to be ≥18 years of age, have a first-ever AIS confirmed by magnetic resonance imaging, and present with significant gait impairment, defined as a Functional Ambulation Classification (FAC) score ≤3. Patients were excluded if they had received antibiotics within the preceding 3 months, used probiotics more than once per week, or had coexisting gastrointestinal disorders (e.g., gastrointestinal infection, ulcerative colitis, or peptic ulcer disease). Additional exclusion criteria included severe comorbid conditions (e.g., malignancy, heart failure, renal failure, or severe hepatic dysfunction) and other disabling conditions affecting lower limb mobility, such as severe osteoarthritis or marked spasticity.

After baseline assessment and rehabilitation safety screening by trained physiotherapists, participants were assigned to either the intervention group (IG, n=24) or control group (CG, n=23). The IG received RAGT (Fourier AL800), while the CG received conventional gait training. Both groups additionally underwent standardized conventional physiotherapy, including lower limb stretching, active movement facilitation, assisted and resistance training, motor control exercises, and standing balance training. The intervention period lasted 4 weeks, consisting of two training sessions per day, five days per week, for a total of 40 sessions. Fecal samples were collected after completion of the intervention, and functional outcomes were evaluated 90 days after stroke onset.

### Clinical and sample data collection

Fresh fecal samples were collected at the end of the 4-week intervention and immediately aliquoted and stored at −80 °C until microbial analysis. Demographic and clinical data were obtained from electronic medical records, including age, sex, smoking status, alcohol consumption, hypertension status, and metabolic parameters (total cholesterol, triglycerides, HDL-C, LDL-C, blood glucose, and glycated hemoglobin [HbA1c]). Immunoinflammatory markers, including leukocyte and lymphocyte counts, were also recorded.

Stroke severity at admission was assessed using the National Institutes of Health Stroke Scale (NIHSS). An NIHSS score ≤4 was defined as mild stroke, >4 as moderate stroke, and >15 as severe stroke. Functional outcomes were assessed using the Barthel Index (BI) and modified Rankin Scale (mRS) at admission and 90 days post-stroke. BI improvement (BI^+^) was calculated as the change in BI score from baseline to follow-up. Favorable outcome was defined as an mRS score of 0–2, and unfavorable outcome as an mRS score of 3–6. Gait impairment was evaluated using the Functional Ambulation Classification (FAC).

### 16S rRNA sequencing and microbiome analysis

Total genomic DNA was extracted from fecal samples, and primers targeting conserved regions of the bacterial 16S rRNA gene were used for PCR amplification. Amplicons were purified, quantified, and pooled to construct sequencing libraries, which were sequenced on the Illumina NovaSeq 6000 platform. Raw sequencing data were generated in FASTQ format.

Bioinformatic processing was conducted using QIIME 2 (Bolyen et al., 2019), including quality control, feature identification, operational taxonomic unit (OTU) clustering, and taxonomic assignment. Alpha diversity was evaluated using ACE, Chao1, PD_whole_tree, Shannon, and Simpson indices. Beta diversity was assessed using principal coordinate analysis (PCoA) based on UniFrac distance matrices. Differential taxa between groups were identified using linear discriminant analysis effect size (LEfSe). Relative abundance profiles and LDA score distributions were used to visualize group differences. Predicted functional profiling of the gut microbiota was predicted using Phylogenetic Investigation of Communities by Reconstruction of Unobserved States 2 (PICRUSt2) and Functional Annotation of Prokaryotic Taxa (FAPROTAX) (Louca et al., 2016; Douglas et al., 2020).

### Statistical analyses

Statistical analyses were performed using R software (version 4.2.1). Continuous variables were compared using Welch’s two-sample *t* test or the Mann–Whitney *U* test. Categorical variables were analyzed using the chi-square test or Fisher’s exact test. Statistical significance was defined as a two-sided *p* value <0.05 or an adjusted *p* value <0.05.

## Results

### RAGT improves functional outcomes after stroke

A total of 47 patients with AIS were included in the final analysis, with 23 patients assigned to the conventional gait training group (CG) and 24 to the RAGT group (IG). Baseline demographic characteristics, including age, sex, smoking status, alcohol consumption, and history of hypertension, did not differ significantly between groups. There were also no significant differences in baseline neurological severity or functional status, as assessed by the NIHSS, FAC, Barthel Index (BI), and modified Rankin Scale (mRS). In addition, metabolic parameters related to AIS pathophysiology (fasting plasma glucose, HbA1c, triglycerides, HDL-C, LDL-C, and total cholesterol) and hematological inflammatory indicators (leukocyte and lymphocyte counts) were comparable between the two groups.

At 90 days post-stroke, patients in the IG demonstrated significantly greater improvement in BI scores (BI^+^) compared with those in the CG. Furthermore, a significantly higher proportion of IG patients achieved favorable functional outcomes (mRS 0–2) than CG patients (87.5% vs. 60.9%). These findings indicate that RAGT was associated with superior functional recovery at 90 days after stroke (**Table 1**).

**Table 1:** Comparison of clinical characteristics between IG and CG patients.

Item	CG N = 23 (49%)*1*	IG N = 24 (51%)*1*	p value*2*
Age(years)	59.57 (13.15)	62.71 (11.75)	0.393
Gender			0.286
male	20 (86.96%)	17 (70.83%)	
female	3 (13.04%)	7 (29.17%)	
Smoking	16 (69.57%)	9 (37.50%)	0.056
Drinking	12 (52.17%)	9 (37.50%)	0.473
HP	13 (56.52%)	12 (50.00%)	0.876
NIHSS (admission)			0.409
≤4	15 (65.22%)	19 (79.17%)	
5-15	7 (30.43%)	5 (20.83%)	
>15	1 (4.35%)	0 (0.00%)	
FAC (admission)			0.451
0	3 (13.04%)	2 (8.33%)	
1	3 (13.04%)	4 (16.67%)	
2	9 (39.13%)	5 (20.83%)	
3	8 (34.78%)	13 (54.17%)	
BI (admission)	80.00 [60.00, 90.00]	75.00 [62.50, 86.25]	0.797
MRS (admission)			0.904
3	13 (56.52%)	15 (62.50%)	
≥3	10 (43.48%)	9 (37.50%)	
glucose	5.93 [5.52, 10.03]	6.08 [5.74, 7.05]	0.848
HbA1c	6.50 [5.70, 10.30]	6.45 [6.30, 6.70]	0.773
TG	4.37 (0.90)	4.33 (0.96)	0.890
HDL-C	1.12 (0.28)	1.25 (0.27)	0.108
LDL-C	2.50 (0.65)	2.54 (0.81)	0.857
TC	1.57 [1.32, 2.32]	1.50 [1.11, 1.97]	0.389
leukocyte	7.32 [6.56, 9.37]	7.44 [5.60, 8.96]	0.295
lymphocyte	2.05 [1.18, 2.66]	1.70 [1.39, 2.08]	0.678
Outcome			
BI+(90 days)	6.52 (16.61)	20.42 (18.88)	0.030
MRS (90 days)			0.049
<3	14 (60.87%)	21 (87.50%)	
≥3	9 (39.13%)	3 (12.50%)	
Recurrence	3 (13.04%)	4 (16.67%)	>0.999

*1* Mean (SD); Median [IQR]; n (%).

*2* Welch Two Sample t test; Mann–Whitney U test; Fisher’s exact test; Pearson’s Chi-squared test.

HP, hypertension; TG, triglyceride; HDL-C, high-density lipoprotein cholesterol; TC, total cholesterol; HbAlc, glycosylated hemoglobin.

### RAGT enhances gut microbiota diversity

**Figure 1:**
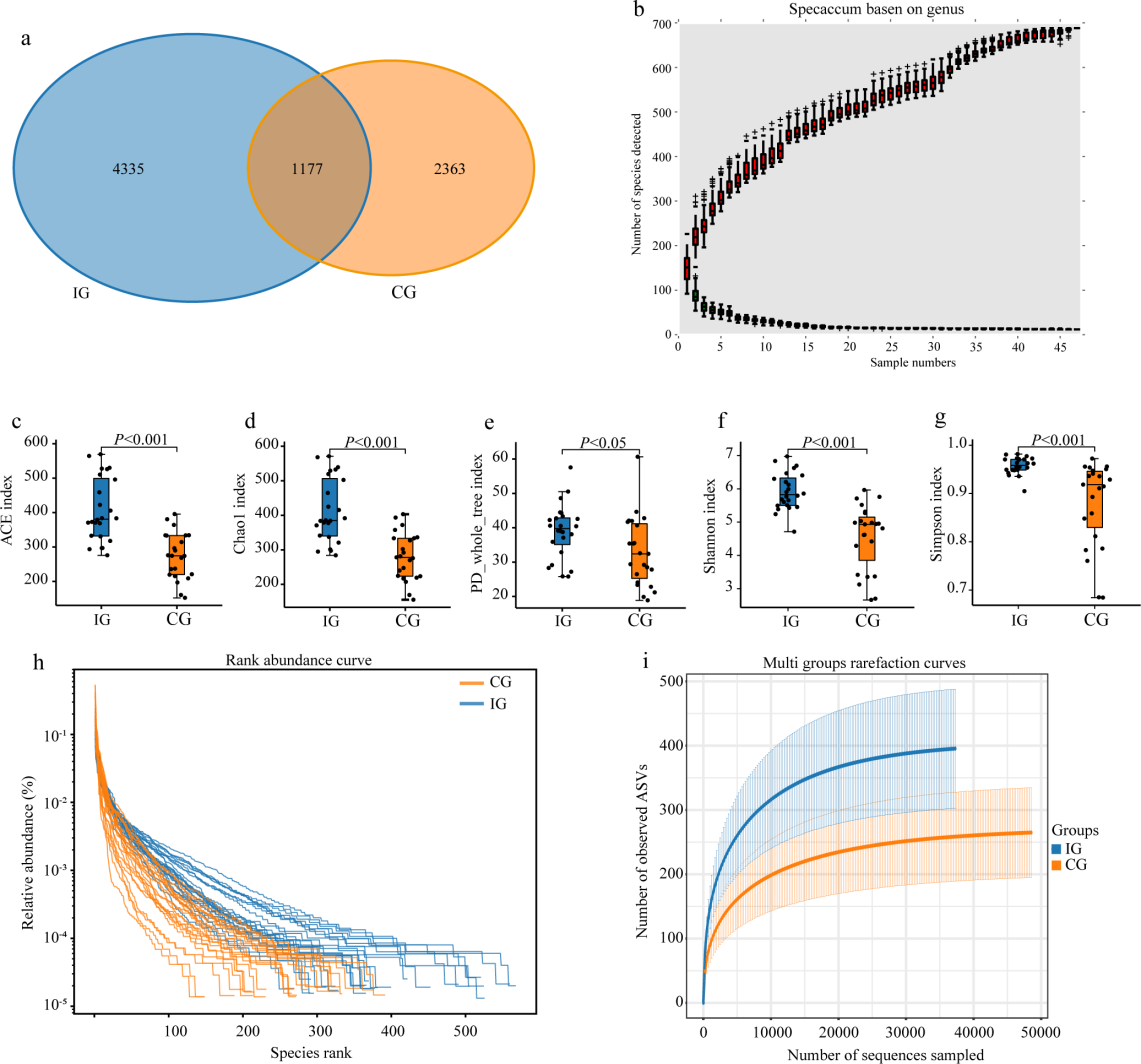
RAGT improved the diversity of the gut microbiota. **(a)** Venn diagram of the features (OTUs) between the two groups; **(b)** genus-level species accumulation curve; alpha diversity of the gut microbiota, including the **(c)** ACE index, **(d)** Chao1 index, **(e)** PD_whole_tree index, **(f)** Shannon index, and **(g)** Simpson index; **(h)** rank abundance curve of the gut microbiota; and **(i)** rarefaction curve of the gut microbiota.

Gut microbiota diversity was assessed using multiple alpha-diversity indices. Venn diagram analysis revealed 1,177 shared operational taxonomic units (OTUs) between groups, with a greater number of unique OTUs observed in the IG (4,335) compared with the CG (2,363) (**Figure 1a**). Species accumulation curves at the genus level reached a plateau, indicating sufficient sequencing depth and reliable sampling (**Figure 1b**).

Alpha-diversity analysis demonstrated significantly higher ACE, Chao1, PD_whole_tree, Shannon, and Simpson indices in IG patients compared with CG patients, indicating increased microbial richness and evenness following RAGT (**Figures 1c-g**). Consistently, rank-abundance curves showed a broader distribution and greater evenness of microbial taxa in the IG (**Figure 1h**). Rarefaction curves further confirmed adequate sequencing depth and persistent differences in gut microbial diversity between groups (**Figure 1i**). Collectively, these results suggest that RAGT markedly improved gut microbiota richness and diversity in stroke patients.

### RAGT reshapes gut microbiota community structure

**Figure 2:**
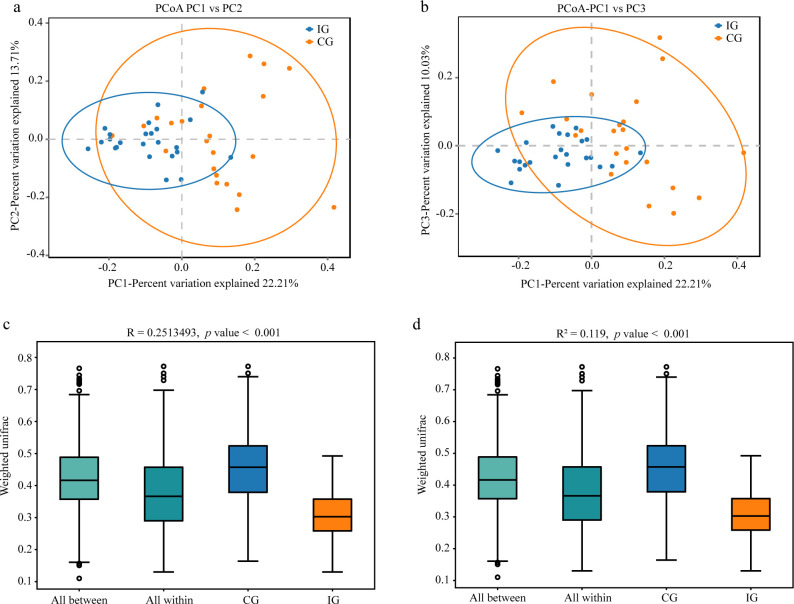
Similarity analysis of the gut microbiota between the two groups. **(a)** Weighted version of the UniFrac-based PCoA plot with PC1 and PC2 at the OTU level among the IG and the CG. X: first principal component, Y: second principal component. **(b)** Weighted version of UniFrac-based PCoA plot with PC1 and PC3 at the OTU level among the IG and the CG. X: first principal component, Y: third principal component. Alpha diversity of gut microbiota including **(c)** the PERMANOVA test based on the weighted UniFrac distance and **(d)** the ANOSIM test based on the weighted UniFrac distance.

To evaluate differences in overall microbial community structure, beta-diversity analysis was performed using weighted UniFrac distance metrics. Principal coordinate analysis (PCoA) based on PC1 and PC2 showed partial overlap between groups, with IG samples clustering within the broader CG distribution (**Figure 2a**). When PC1 and PC3 were considered, IG samples formed a more distinct and compact cluster compared with CG samples, which displayed greater interindividual variability (**Figure 2b**). These findings indicate increased homogeneity of gut microbiota composition in IG patients following RAGT. Statistical validation using PERMANOVA and ANOSIM confirmed significant differences in microbial community structure between the IG and CG (**Figures 2c, d**).

### RAGT modulates gut microbiota composition

**Figure 3:**
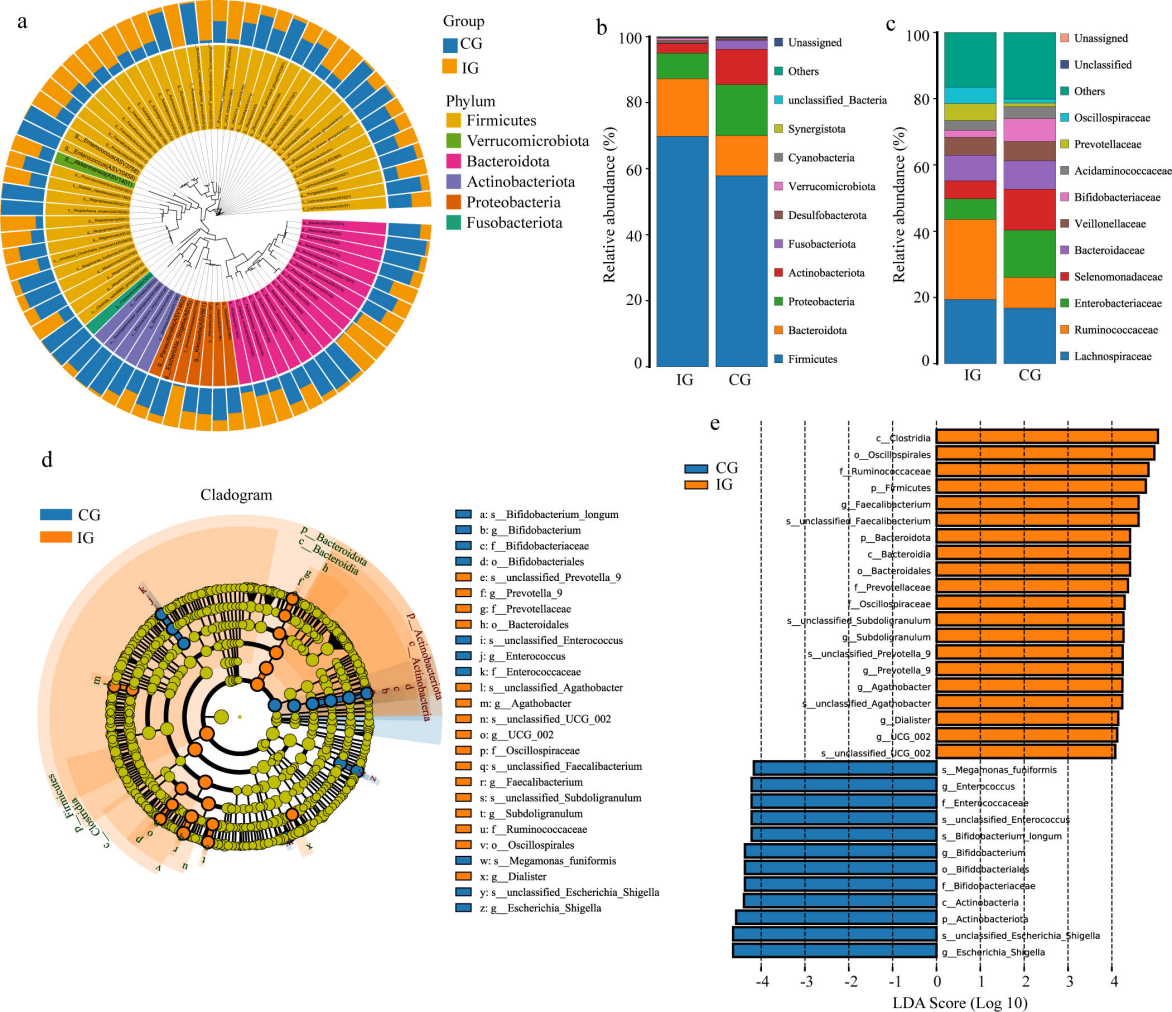
Comparison of the gut microbiota composition between the two groups. **(a)** Sample community distribution map based on the phylogenetic tree. The relative abundance of the gut microbiota at the phylum **(b)** and family **(c)** levels. **(d)** LEfSe analysis cladogram diagram of the gut microbiota between the IG and CG. **(e)** LDA score distribution histogram of the gut microbiota between the IG and CG, |LDA score| > 4.

Taxonomic profiling revealed that *Bacteroidota*, *Firmicutes*, *Proteobacteria*, and *Actinobacteria* were the dominant phyla in both groups (**Figure 3a**; **Supplementary Table 1**). Compared with the CG, the IG exhibited a higher relative abundance of *Firmicutes* and *Bacteroidota*, accompanied by a reduction in *Proteobacteria*, *Actinobacteria*, and *Fusobacteria* (**Figure 3b**). At the family level, RAGT was associated with significant enrichment of *Lachnospiraceae*, *Ruminococcaceae*, and *Oscillospiraceae*, taxa commonly linked to short-chain fatty acid production and gut homeostasis. In contrast, the relative abundances of *Enterobacteriaceae* and *Bifidobacteriaceae* were significantly reduced in the IG (**Figure 3c**). These compositional changes were further supported by phylogenetic tree–based community distribution analysis. LEfSe analysis identified specific discriminatory taxa between groups. RAGT significantly increased the abundance of beneficial genera, including *Prevotella*, *Agathobacter*, *Faecalibacterium*, *Subdoligranulum*, *Dialister*, and *UCG_002*, while reducing *Bifidobacterium*, *Enterococcus*, and *Escherichia_Shigella* (**Figures 3d, e**).

**Figure 4:**
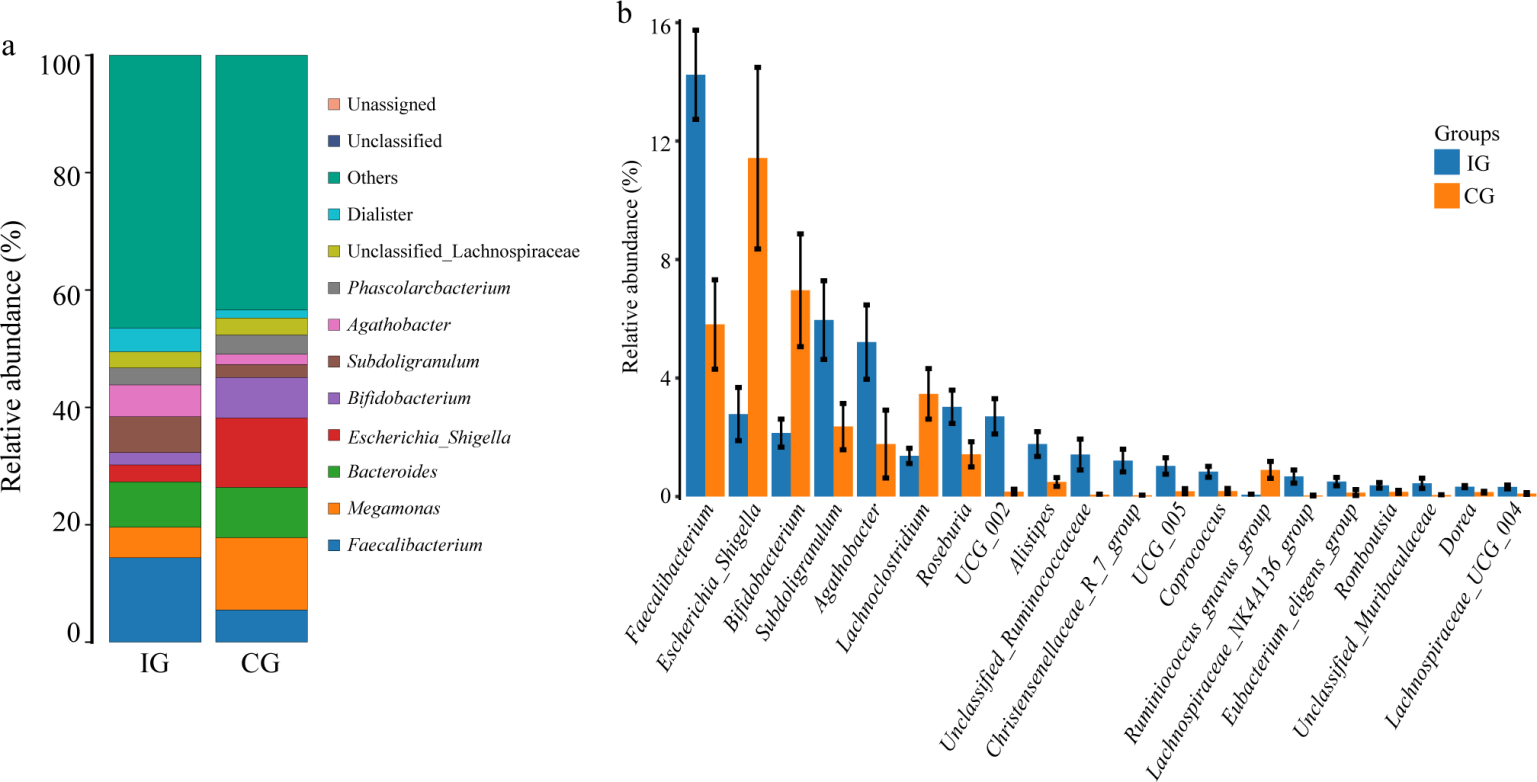
Differences in the gut microbiota at the genus and species levels between the two groups. **(a)** The relative abundance of the gut microbiota at the genus level. **(b)** Statistical analysis of the gut microbiota at the genus level through ANOVA.

Quantitative genus-level analysis demonstrated marked increases in *Faecalibacterium*, *Subdoligranulum*, *Agathobacter*, and *Roseburia* in the IG (**Figures 4a, b**). Specifically, the relative abundance of *Faecalibacterium* increased from 5.81 ± 1.44% to 14.24 ± 1.36%, *Subdoligranulum* from 2.36 ± 0.37% to 5.57 ± 0.65%, and *Agathobacter* from 1.73 ± 1.02% to 5.21 ± 1.39%. Conversely, certain genera were significantly reduced in the IG, including *Escherichia_Shigella* (11.42 ± 2.71% to 2.78 ± 1.04%), *Bifidobacterium* (6.96 ± 2.09% to 2.14 ± 0.62%), and *Lachnoclostridium* (3.46 ± 0.49% to 1.37 ± 0.39%). Taken together, these results suggested that RAGT modulates gut microbiota composition.

### RAGT improves predicted gut microbiota function

**Figure 5:**
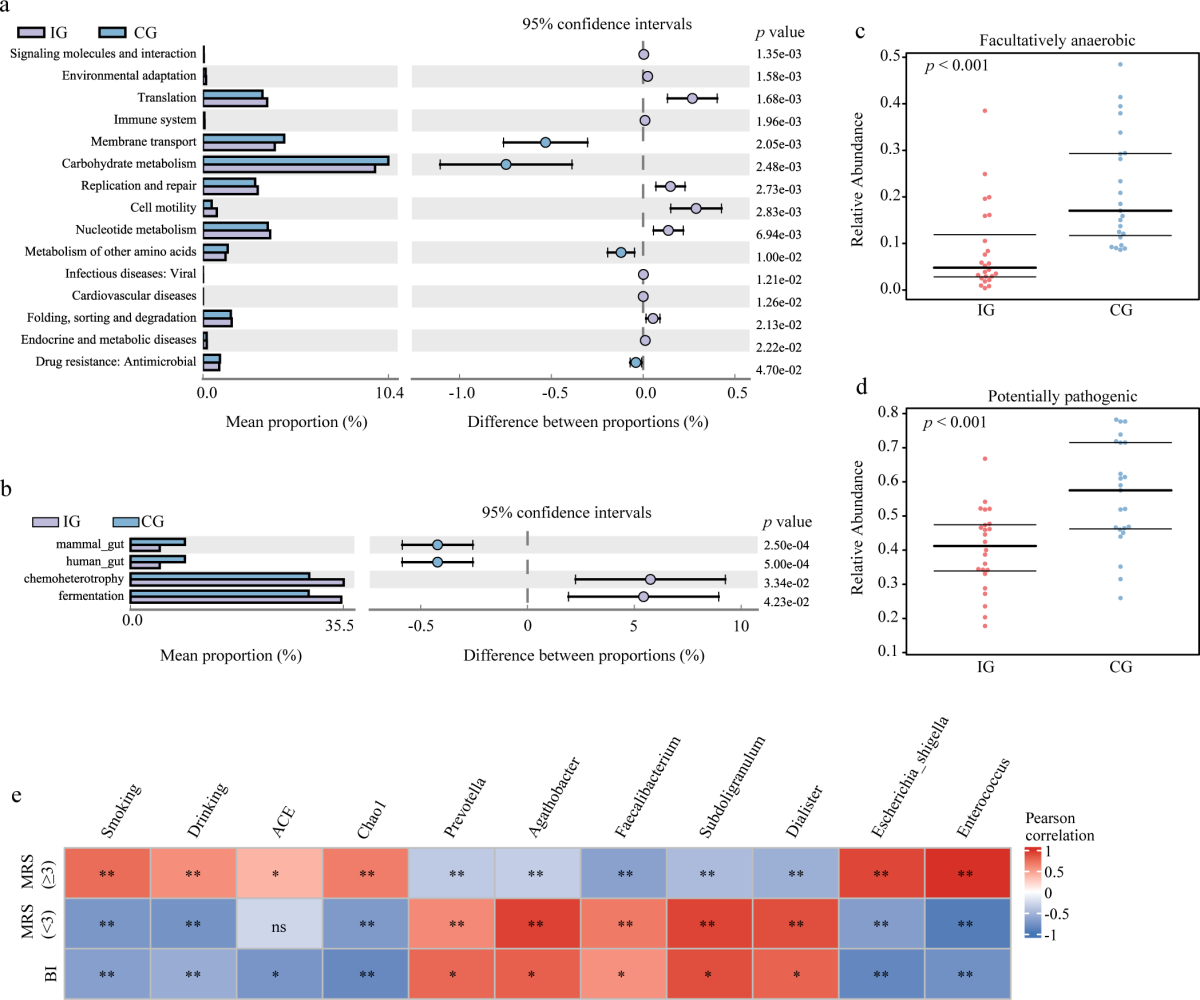
RAGT therapy improved gut microbiota function. **(a)** KEGG pathways inferred from the gut microbiota via PICRUSt2. **(b)** Ecological functional prediction inferred from the gut microbiota via FAPROTAX. **(c)** Relative abundance of facultatively anaerobic bacteria predicted by BugBase. **(d)** Relative abundance of potentially pathogenic bacteria predicted by BugBase. **(e)** The correlation among BI, MRS (<3), MRS (≥3) and all other indices calculated by Person; ns, no significance, *p < 0.05, **p < 0.01.

Functional prediction using PICRUSt2 revealed distinct metabolic shifts in the gut microbiota of IG patients. Compared with the CG, pathways related to carbohydrate metabolism, membrane transport, and metabolism of other amino acids were reduced, whereas pathways involved in translation, replication and repair, nucleotide metabolism, and cell motility were enriched in the IG (**Figure 5a**). FAPROTAX analysis further indicated increased representation of microbial functions associated with fermentation and chemoheterotrophy in the IG (**Figure 5b**). BugBase phenotype prediction demonstrated a lower abundance of facultatively anaerobic bacteria and a significantly reduced pathogenic potential in the gut microbiota of IG patients (**Figures 5c, d**). Correlation analysis showed that improvements in functional outcomes (BI increase and favorable mRS scores) were significantly associated with changes in gut microbiota composition and predicted functional indices (**Figure 5e**), suggesting a close link between RAGT-induced microbiota modulation and post-stroke recovery.

## Discussion

In this prospective study, we demonstrated that RAGT was associated with both improved functional recovery and partial restoration of stroke-induced gut microbiota dysbiosis in patients with AIS. Compared with conventional gait training, RAGT increased gut microbial diversity, enriched beneficial commensal taxa, reduced opportunistic pathogens, and improved microbial functions predicted by gut microbiota. Importantly, patients receiving RAGT achieved superior functional outcomes at 90 days post-stroke, suggesting that modulation of the gut microbiota may represent a previously underappreciated mechanism contributing to the therapeutic effects of robotic rehabilitation.

Accumulating evidence supports the concept of bidirectional communication between the gut and the brain through neural, neuroendocrine, and immunological pathways, collectively referred to as the gut–brain axis (Bravo et al., 2011; Carabotti et al., 2015; O'mahony et al., 2015; Winek et al., 2016; Durgan et al., 2019; Luan et al., 2019). Under physiological conditions, gut microbes are spatially segregated from the intestinal epithelium by a mucus layer and contribute to host homeostasis through the production of bioactive metabolites, including short-chain fatty acids (SCFAs), vitamins, and immunomodulatory compounds (Atarashi et al., 2015). Ischemic stroke disrupts this balance, leading to impaired intestinal barrier integrity, reduced mucus secretion, gut microbiota dysbiosis, and translocation of microbial products into the systemic circulation (Houlden et al., 2016; Singh et al., 2016; Stanley et al., 2016; Xia et al., 2019). These changes promote systemic inflammation and neuroinflammation, thereby exacerbating brain injury and impairing neurological recovery.

Consistent with previous reports, stroke-induced gut dysbiosis is characterized by reduced microbial diversity, altered Firmicutes-to-Bacteroidetes ratios, expansion of opportunistic pathogens, and depletion of beneficial commensals (Yin et al., 2015; Houlden et al., 2016; Li et al., 2019; Tan et al., 2021). Therapeutic strategies aimed at restoring gut microbiota homeostasis, such as antibiotics, prebiotics, probiotics, dietary modulation, and fecal microbiota transplantation (FMT), have shown neuroprotective effects in experimental stroke models (Duncan and Flint, 2013; Li et al., 2020; Olas, 2020). These findings underscore the importance of gut microbiota modulation as a potential therapeutic avenue in stroke rehabilitation.

Exercise is increasingly recognized as a potent non-pharmacological modulator of gut microbiota composition and function predicted by gut microbiota. Both animal and human studies indicate that moderate exercise enhances intestinal barrier integrity, increases microbial diversity, enriches SCFA-producing bacteria, and promotes a shift toward an anti-inflammatory immune milieu (Martins et al., 2008; Keirns et al., 2020; Scheffer and Latini, 2020; Aragón-Vela et al., 2021). In athletes, gut microbiota diversity is markedly higher than in sedentary individuals, and exercise interventions following myocardial infarction have been shown to reverse disease-associated reductions in microbial gene richness (Zhou et al., 2022). However, evidence regarding the effects of structured poststroke rehabilitation, particularly gait training, on gut microbiota dysbiosis in humans remains limited.

Patients with acute stroke and lower limb dysfunction often experience prolonged immobility, impaired bowel motility, and delayed mobilization. RAGT systems, equipped with body-weight support and exoskeletal assistance, allow earlier, safer, and more intensive walking practice than conventional therapy. Such early mobilization may directly influence gastrointestinal function and indirectly reshape the gut microbial ecosystem. Based on these considerations, we hypothesized that RAGT could reverse stroke-induced gut dysbiosis and thereby contribute to improved neurological recovery.

A key finding of our study was the enrichment of SCFA-producing bacteria following RAGT. Specifically, patients in the intervention group exhibited increased abundances of *Lachnospiraceae*, *Ruminococcaceae*, and *Oscillospiraceae*, as well as higher levels of *Faecalibacterium*, *Agathobacter*, and *Roseburia*. These taxa are well-established producers of SCFAs, particularly butyrate, which play essential roles in maintaining intestinal barrier integrity, modulating immune responses, and regulating neuroinflammation (Chen et al., 2019; Dalile et al., 2019; Vacca et al., 2020; Tangestani et al., 2021).

Notably, *Faecalibacterium* is one of the most abundant and functionally important genera in the human gut, accounting for up to 15% of total microbial abundance. It produces large amounts of butyrate and exerts potent anti-inflammatory effects, locally in the gut (Duncan et al., 2002; Miquel et al., 2013). Reduced abundance of *Faecalibacterium* is widely regarded as a hallmark of gut dysbiosis and has been implicated in multiple inflammatory and neurological disorders. Similarly, *Roseburia* and *Agathobacter* are major contributors to SCFA production and have been linked to metabolic and cardiovascular health (Rosero et al., 2016; Tamanai-Shacoori et al., 2017; Kasahara et al., 2018; Liu et al., 2019). The coordinated enrichment of these taxa observed in our study suggests that RAGT promotes a metabolically favorable gut environment conducive to neurological recovery.

In parallel with the enrichment of beneficial taxa, RAGT was associated with a reduction in opportunistic pathogens, particularly members of the *Enterobacteriaceae* family, including *Escherichia_Shigella*. Overgrowth of *Enterobacteriaceae* following stroke has been consistently reported and is closely associated with increased lipopolysaccharide (LPS) production, systemic inflammation, and worsened neurological outcomes (Xu et al., 2021). Circulating LPS and proinflammatory cytokines can penetrate the compromised blood–brain barrier, activate microglia, and exacerbate neuronal damage (Fenton and Golenbock, 1998; Benakis et al., 2016; Klimiec et al., 2016; Singh et al., 2016; Brown, 2019; Kim et al., 2020). Our findings of reduced *Enterobacteriaceae* abundance and lower predicted pathogenic potential in the intervention group suggest that RAGT may attenuate stroke-induced systemic inflammation by suppressing pathogenic microbial expansion.

Several limitations of this study should be acknowledged. First, fecal samples were collected only after the intervention, precluding longitudinal analysis of microbiota dynamics before and during rehabilitation. Second, functional inferences were based on predictive bioinformatic tools rather than direct metabolomic or transcriptomic measurements. Third, the relatively modest sample size may limit statistical power and the ability to perform subgroup or multivariable analyses. Future studies incorporating longitudinal sampling, multi-omics approaches, and larger cohorts are warranted to further elucidate the causal relationships between rehabilitation, gut microbiota, and stroke recovery.

## Conclusion

Our findings suggest that RAGT improves functional outcomes after stroke, which is associated with alteration of gut microbiota homeostasis by enriching SCFA-producing bacteria and suppressing opportunistic pathogens. These microbiota-associated changes may represent an important biological mechanism underlying the neurorehabilitative benefits of RAGT and support the integration of microbiota-focused perspectives into poststroke rehabilitation strategies.

## Data Availability

The datasets presented in this study can be found in online repositories. The names of the repository/repositories and accession number(s) can be found in the article/**Supplementary Material**.
